# Impact of early decompression surgery and injury characteristics on neurological recovery following traumatic cervical spinal cord injury

**DOI:** 10.1016/j.bas.2025.104326

**Published:** 2025-07-10

**Authors:** Ngo Dinh Trung, Hoang Xuan Su, Dao Trong Chinh, Nguyen Chi Tam, Le Nam Khanh, Do Van Nam

**Affiliations:** a Department of Surgical and Transplant Intensive Care Unit, Military Central Hospital 108, Viet Nam; b Institute of Biomedicine and Pharmacy, Vietnam Military Medical University, HanoiViet Nam

**Keywords:** Cervical spinal cord injury, Neurological recovery, Early decompression surgery, Spinal cord injury length, Prognostic factors

## Abstract

**Introduction:**

Traumatic cervical spinal cord injury (SCI) frequently leads to severe neurological deficits. Early decompressive surgery, when performed shortly after the injury, is believed to improve outcomes. However, the impact of surgical timing, calculated from the moment of injury, and injury characteristics on neurological recovery remains poorly understood.

**Research question:**

This study aims to assess the impact of early decompressive surgery and injury characteristics on neurological recovery in traumatic cervical SCI patients.

**Material and methods:**

A retrospective case-control study was conducted at the 108 Central Military Hospital in Hanoi, Vietnam, between January 2018 and June 2023. Data collected included demographics, injury characteristics, time from injury to decompression surgery, and clinical outcomes at the 1-year follow-up.

**Results:**

Among the 193 patients initially screened, 156 met the inclusion criteria. Neurological recovery was observed in 44.2 % of patients. Early decompression within 24 h significantly improved recovery outcomes (OR = 3.12, p = 0.006). Injury at C1–C3 levels, longer spinal cord injury length, and severe spinal canal stenosis were associated with poorer recovery (OR = 0.34, p = 0.015; OR = 0.21, p < 0.001; OR = 0.34, p = 0.009, respectively). Prolonged ICU stay correlated with worse recovery (OR = 0.90, p = 0.002).

**Discussion and conclusion:**

Early decompressive surgery, thorough assessment of injury characteristics, and minimizing ICU stay duration are critical for improving neurological recovery. These findings highlight the importance of timely surgery and strategic clinical management in enhancing recovery outcomes in traumatic cervical SCI patients.

## Highlights

• Early decompressive surgery performed within 24 h significantly improves neurological recovery in patients with traumatic cervical spinal cord injury (SCI).
• Injury characteristics such as spinal cord injury at the C1–C3 level, longer spinal cord injury length, and severe spinal canal stenosis are associated with poorer recovery outcomes.
• Prolonged ICU stay is also a significant factor that reduces the likelihood of recovery.
• Identified prognostic factors, including surgical timing and injury characteristics, can guide clinical strategies to optimize recovery and patient outcomes after SCI.

## Abbreviations:

AIS: (American spinal cord injury association impairment scale)
CI: (confidence interval)
ICU: (intensive care unit)
IQR: (interquartile range)
MRI: (magnetic resonance imaging)
OR: (odds ratio)
RR: (relative risk)
SCI: (spinal cord injury)
T2WI: (T2-weighted imaging)

## 1 Introduction

Traumatic spinal cord injury (SCI) presents a significant global health challenge, with a consistently high incidence of between 20 and 45 cases per million people from 2000 to 2021 (Lu et al., 2024). This injury is particularly prevalent in developing countries, where the leading causes, such as motor vehicle accidents and falls, are more frequent (Covell et al., 2024). Among the various types of SCI, traumatic cervical SCI often results in severe neurological deficits, including motor, sensory, and autonomic dysfunction (Nekhlopochyn et al., 2024). Therefore, it not only drastically diminishes patients' quality of life but also imposes substantial social and economic burdens on healthcare systems (Merritt et al., 2019). Additionally, traumatic cervical SCI is associated with a high incidence of respiratory complications and a notable reduction in life expectancy, highlighting the urgent need for effective treatment and rehabilitation strategies (Michel-Flutot et al., 2023; Ko, 2023).

In the management of SCI, early surgical intervention, particularly decompressive surgery, has shown promise in improving neurological outcomes (Fehlings et al., 2024; Vasquez-Paredes et al., 2024). Previous studies indicated that early decompression significantly enhances recovery, as it helps mitigate secondary injury mechanisms like ischemia and inflammation (Franchyeda, 2024; Jin et al., 2024; Kwon et al., 2024). However, the intricate relationship between the timing of surgery and specific injury characteristics, such as lesion length, spinal canal stenosis, and injury location, warrants further investigation to better understand their impact on neurological recovery.

This study aims to examine the impact of early decompressive surgery and specific injury characteristics on neurological recovery in patients with traumatic cervical SCI. By identifying robust prognostic factors, we hope to provide insights that can guide clinical decision-making, optimize long-term patient outcomes, and inform the development of targeted rehabilitation strategies for traumatic cervical SCI patients.

## 2 Methods

### 2.1 Study design and patient selection

**Fig. 1:**
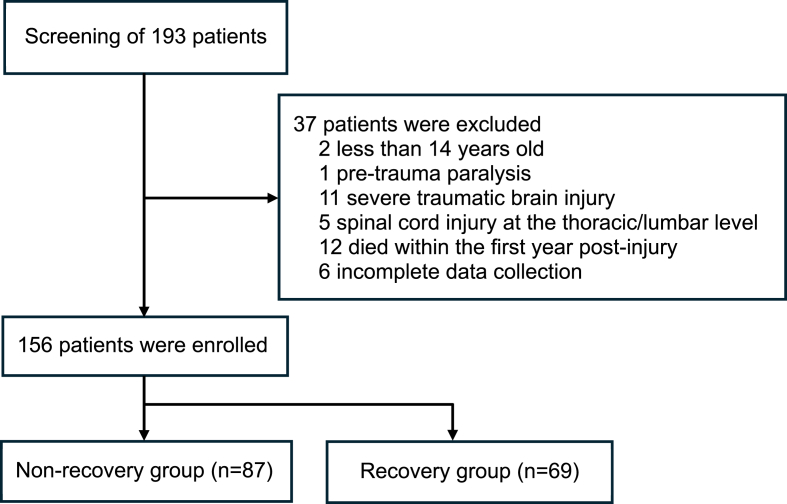
The flow diagram of patient selection in the study.

This was a single-center, retrospective, case-control study conducted to assess the recovery of neurological function following traumatic cervical spinal cord injury. The study was carried out at the 108 Central Military Hospital, Hanoi, Vietnam, involving patients admitted between January 2018 and June 2023. Inclusion criteria included patients aged ≥14 years at the time of injury, with a follow-up duration of at least 1-year post-injury, and those who experienced upper and/or lower limb paralysis following the injury. Exclusion criteria included patients with a history of pre-existing paralysis, severe concomitant traumatic brain injury, lesions in spinal regions other than the cervical spinal cord (e.g., thoracic or lumbar injuries), those who died within the first year after injury, or those for whom sufficient data could not be collected. The flow diagram showing patient selection is presented in Fig. 1. Patients were provided with a detailed explanation of the study's purpose and signed informed consent before participation. This study was approved by the local ethics committee, and was conducted in accordance with institutional and national guidelines.

### 2.2 Data collection

Data at the time of admission and during hospitalization were collected from the patient's electronic medical records, and the 1-year post-trauma status was obtained through patient follow-up visits at the 108 Central Military Hospital. The following variables were recorded: age, sex, mechanism of spinal cord injury, need for vasopressor support, and duration of intensive care unit (ICU) stay. Neurological function was assessed at two time points: upon hospital admission and at the 1-year follow-up. Evaluation was performed using the American Spinal Injury Association Impairment Scale (AIS), in accordance with the International Standards for Neurological Classification of Spinal Cord Injury (ISNCSCI) (Kirshblum et al., 2011). The AIS grade classification is based on a standardized neurological examination, including assessments of motor strength in key muscle groups and sensory function (light touch and pinprick) across dermatomes, enabling assignment to one of five AIS grades (A–E), where grade A represents complete injury and grade E denotes normal neurological status (Kirshblum et al., 2011). Imaging data included the highest level of spinal cord injury on magnetic resonance imaging (MRI), the degree of spinal canal stenosis assessed using the Torg-Pavlov ratio, length of spinal cord injury, and increased signal on T2-weighted imaging (T2WI) (Yue et al., 2001). Surgical treatment was defined as any cervical spine surgery performed during the index hospitalization due to trauma-related cervical spinal injury. This included procedures such as decompression, stabilization, or alignment correction, as documented in operative records.

### 2.3 Outcomes

The primary outcome was neurological recovery, defined as an improvement of at least one AIS grade from admission to the 1-year follow-up. Secondary outcomes included neuropathic pain and bladder/bowel dysfunction at 1 year post-injury. Neuropathic pain was defined as persistent pain with typical neuropathic features (e.g., burning, shooting, electric shock-like) reported by the patient and confirmed through clinical evaluation (Widerström-Noga et al., 2014). Bladder/bowel dysfunction was defined as persistent neurogenic lower urinary tract or bowel disorder at 1 year post-injury. This included urinary retention or incontinence requiring intermittent catheterization or pharmacologic therapy, and bowel dysfunction necessitating manual evacuation or bowel management programs. At follow-up, dysfunction was assessed through patient-reported symptoms, review of continence history, and structured neurological examination, including anal tone, perianal sensation, and voluntary anal contraction (Krogh et al., 2017; Biering-Sørensen et al., 2018).

### 2.4 Management of SCI in the acute setting

At our center, patients with cervical spinal cord injury are managed following a standardized acute care protocol. This includes immediate cervical spine immobilization to prevent further mechanical injury. Hemodynamic stabilization is initiated early, aiming to maintain a mean arterial pressure of at least 75–80 mmHg but not exceeding 90 mmHg, in order to optimize spinal cord perfusion and minimize secondary injury. When indicated, surgical decompression and stabilization are performed, preferably within 24 h, to alleviate spinal cord compression and restore spinal alignment.

During the acute phase, patients are admitted to the intensive care unit for continuous neurological monitoring and comprehensive supportive care, including respiratory management, infection prevention, pressure ulcer prophylaxis, and preparation for rehabilitation. This management approach aligns with current evidence-based recommendations for acute SCI care (Fehlings et al., 2017).

### 2.5 Statistical analysis

Continuous variables were presented as medians (interquartile ranges), including age, length of SCI, and ICU stay duration, as these variables did not follow a normal distribution (Shapiro–Wilks p-value <0.05). Categorical variables were presented as frequencies (percentages). The Wilcoxon signed-rank test was used to assess changes in AIS grade between the 1-year follow-up and the admission time point. The chi-square test for categorical variables and the Mann-Whitney *U* test for continuous variables were used to assess differences in various characteristics between the neurological recovery and non-recovery groups. Univariable and multivariable logistic regression analyses were performed to identify predictors of neurological function improvement. Data analysis was performed using SPSS software version 27.0 (IBM, New York, USA), with a significance threshold of p < 0.05.

## 3 Results

A total of 193 patients were screened for the study, of which 37 were excluded based on predefined criteria. The remaining 156 patients met the eligibility criteria and were included in the study. These patients were then categorized into two groups: the non-recovery group (87 patients), accounting for 55.8 %, and the recovery group (69 patients), accounting for 44.2 % (Fig. 1).

### 3.1 Changes in upper and lower extremity muscle strength

**Fig. 2:**
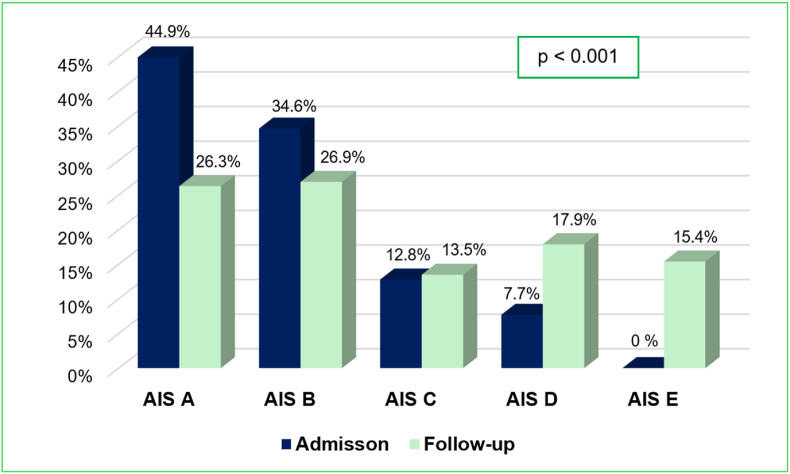
**Improvement in AIS grade between the 1-year follow-up and the admission time point.** AIS, American Spinal Injury Association Impairment Scale.

There was a significant improvement in neurological function as measured by AIS grade one year after cervical spinal cord injury compared to the admission time point (p < 0.001). The proportion of patients with complete injury (AIS A) decreased from 44.9 % at admission to 26.3 % at the one-year follow-up, while the percentage of patients classified as normal neurological function (AIS E) increased from 0 % to 15.4 %. Similarly, the proportion of patients in AIS D, representing functional motor recovery, rose from 7.7 % to 17.9 %. These findings suggest meaningful neurological recovery in a subset of patients over the one-year period (Fig. 2).

### 3.2 Clinical and MRI characteristics comparison between the two groups

Table 1 presents the clinical differences between the two groups (recovery and non-recovery) in terms of neurological recovery after traumatic cervical spinal cord injury. The recovery group had higher proportions of patients with AIS grade C or D on admission. The recovery group also had a shorter median ICU stay (4 days vs. 5 days, p = 0.017) and lower vasopressor use (33.3 % vs. 58.6 %, p = 0.002). Early surgery within 24 h after injury was more common in the improve group (65.2 % vs. 49.4 %, p = 0.048). In contrast, the non-improve group had higher rates of neuropathic pain (55.2 % vs. 39.1 %, p = 0.046) and bowel/bladder dysfunction (67.8 % vs. 29.0 %, p < 0.001) at one year. No significant differences were observed between the two groups in terms of gender, age, cause of injury, or the overall rate of surgical intervention.

**Table 1:** Comparison of clinical characteristics between the two groups.

Variables	Total (n = 156)	Non-recovery (n = 87)	recovery (n = 69)	p-value
Male, n (%)	138 (88.5)	75 (86.2)	63 (91.3)	0.322
Age, median (IQR), year	55 (43–63)	58 (46–65)	54 (40–61)	0.140
Cause of SCI, n (%)				0.051
Fall	63 (40.4)	40 (46.0)	23 (33.3)	–
Occupational accident	26 (16.7)	18 (20.7)	8 (11.6)	–
Traffic accident	52 (33.3)	22 (25.3)	30 (43.5)	–
Other causes	15 (9.6)	7 (8.0)	8 (11.6)	–
AIS grade C or D on admission, n (%)	32 (20.5)	8 (9.2)	24 (34.8)	**< 0.001**
Need for vasopressor, n (%)	74 (47.4)	51 (58.6)	23 (33.3)	**0.002**
Surgical treatment, n (%)	106 (67.9)	57 (65.5)	49 (71.0)	0.465
Early surgery within the first 24 h after injury, n (%)	88 (56.4)	43 (49.4)	45 (65.2)	**0.048**
ICU stay duration, median (IQR), day	5 (1–8)	5 (2–10)	4 (1–7)	**0.017**
Neuropathic pain at 1 year post-trauma, n (%)	75 (48.1)	48 (55.2)	27 (39.1)	**0.046**
Bowel/bladder dysfunction at 1 year post-trauma, n (%)	79 (50.6)	59 (67.8)	20 (29.0)	**< 0.001**

Notes: IQR, interquartile range; SCI, spinal cord injury; AIS, American spinal injury association impairment scale; ICU, intensive care unit. Bold values in the p-value column indicate a statistically significant difference (p < 0.05).

Table 2 compares MRI characteristics of spinal cord injuries between the two groups. The non-recovery group had a higher percentage of severe spinal canal stenosis (43.7 % vs. 21.7 %, p = 0.022) and longer spinal cord injury length (median 28 mm vs. 20 mm, p < 0.001). No significant differences were found between the groups regarding the highest level of spinal cord injury or increased signal on T2-weighted imaging.

**Table 2:** Comparison of spinal cord injury characteristics on MRI between the two groups.

Variables	Total (n = 156)	Non-recovery (n = 87)	Recovery (n = 69)	p value
Highest level of spinal cord injury on MRI, n (%)				0.341
C1-C3	45 (28.8)	29 (33.3)	16 (23.2)	–
C4-C5	92 (59.0)	49 (56.3)	43 (62.3)	–
C6-C7	19 (12.2)	9 (10.4)	10 (14.5)	–
Degree of spinal canal stenosis, n (%)				**0.022**
None	21 (13.5)	8 (9.2)	13 (18.8)	–
Mild	37 (23.7)	17 (19.5)	20 (29.0)	–
Moderate	45 (28.8)	24 (27.6)	21 (30.4)	–
Severe	53 (34.0)	38 (43.7)	15 (21.7)	–
Spinal cord injury length, median (IQR), mm	22 (18–37)	28 (20–41)	20 (15–26)	**<0.001**
Increased signal on T2WI, n (%)	87 (55.8)	53 (60.9)	34 (49.3)	0.146

Notes: MRI, magnetic resonance imaging; IQR, interquartile range; T2WI, T2-weighted Imaging. Bold values in the p-value column indicate a statistically significant difference (p < 0.05).

### 3.3 Prognostic factors for neurological recovery in traumatic cervical SCI

Table 3 presents the prognostic factors for neurological recovery in patients with traumatic cervical spinal cord injury. In the univariate analysis, many factors showed statistical significance. However, the multivariate analysis identified only a few independent factors significantly associated with neurological recovery. Specifically, early surgery performed within 24 h post-injury independently increased the likelihood of neurological recovery by 3.12 times (odds ratio (OR) = 3.12, p = 0.006). Conversely, spinal cord injury at the C1-C3 level independently reduced the chances of recovery by 66 % (OR = 0.34, p = 0.015). Additionally, each 1 cm increase in spinal cord injury length was independently associated with a 79 % reduction in the likelihood of recovery (OR = 0.21, p < 0.001). Severe spinal canal stenosis independently decreased the recovery odds by 66 % (OR = 0.34, p = 0.009). Lastly, each additional day spent in the ICU was independently linked to a 10 % decrease in the probability of neurological recovery (OR = 0.90, p = 0.002).

**Table 3:** Prognostic factors for neurological recovery in patients with traumatic cervical spinal cord injury.

Variable	Logistic regression			
	Univariate		Multivariate (step-down)	
	OR (95 % CI)	p-value	OR (95 % CI)	p-value
Age/10	0.86 (0.69–1.06)	0.159	–	–
AIS grade C or D on admission	5.27 (2.18–12.69)	**<0.001**	–	–
Spinal cord injury C1–C3	0.36 (0.17–0.76)	**0.007**	0.34 (0.14–0.81)	**0.015**
Increased signal on T2WI	0.62 (0.33–1.18)	0.147	–	–
Severe spinal canal stenosis	0.36 (0.18–0.73)	**0.005**	0.34 (0.15–0.76)	**0.009**
Length of spinal cord injury (cm)	0.55 (0.41–0.74)	**<0.001**	0.21 (0.09–0.48)	**<0.001**
Early surgery within 24 h post-injury	1.92 (1.00–3.67)	**0.049**	3.12 (1.38–7.03)	**0.006**
Need for vasopressor	0.35 (0.18–0.68)	**0.002**	–	–
ICU stay duration (days)	0.24 (0.12–0.48)	**<0.001**	0.90 (0.85–0.96)	**0.002**

Notes: OR, odds ratio; CI: confidence interval; UEMS, upper extremity motor score; LEMS, lower extremity motor score; MRC, medical research council; ICU, intensive care unit. Bold values in the p-value column indicate a statistically significant association (p < 0.05).

## 4 Discussion

The primary objective of this study was to identify independent prognostic factors influencing neurological recovery following traumatic cervical spinal cord injuries (SCI). Our analysis identified significant associations between improved neurological outcomes and timing of surgical intervention, spinal cord injury level, lesion length, severity of spinal canal stenosis, and ICU stay duration.

### 4.1 Impact of early decompression surgery on neurological recovery

Among prognostic factors for neurological recovery, early surgical decompression emerged as a key determinant of recovery, a result consistent with previous researches (Fehlings et al., 2024; Vasquez-Paredes et al., 2024; Badhiwala et al., 2021; Sattari et al., 2024). Fehlings et al. established that patients who underwent decompression within 24 h had double the likelihood of experiencing a recovery of AIS ≥2 grades at both 6 months (relative risk (RR): 2.76, 95 % confidence interval (CI) 1.60–4.98) and 12 months (RR: 1.95, 95 % CI 1.26–3.18) compared to those who had decompression after 24 h (Fehlings et al., 2024). Similarly, Vasquez-Paredes et al. observed that early decompression within 24 h led to significant improvements in AIS scores after a 12-month follow-up (Vasquez-Paredes et al., 2024). The mechanism underlying these findings likely involves early intervention preventing further secondary injury, such as edema, inflammation, ischemia, oxidative stress, and apoptosis, creating a more favorable environment for tissue preservation and regeneration (Franchyeda, 2024; Jin et al., 2024; Kwon et al., 2024). Moreover, prompt decompression reduces intramedullary pressure, a critical factor for maintaining neurological function (Lambrechts et al., 2023; Zhu et al., 2023). In our study, the feasibility of early decompression was supported by the institutional capacity of our center. The 108 Military Central Hospital maintains 24/7 availability of neurosurgeons, rapid imaging access (MRI, CT), and streamlined emergency protocols, all of which enable timely surgical intervention and may have contributed to the improved neurological outcomes observed.

### 4.2 Impact of other factors on neurological recovery

Our results corroborate the notion that injuries at the C1–C3 levels are associated with poorer neurological recovery, aligning with prior studies (Futch et al., 2023; Fallah et al., 2023; Khorasanizadeh et al., 2019). Futch et al. reported that upper cervical injuries (C1–C3) are more commonly linked to falls and are also associated with a higher incidence of pre-existing conditions like diabetes, which may further hinder recovery (Futch et al., 2023). Fallah et al. similarly highlighted that significant motor recovery is often observed in lower cervical injuries, whereas recovery in high cervical injuries (C1–C4) is more limited, particularly in AIS A injuries (Fallah et al., 2023). These upper cervical spinal cord injuries are also linked to increased mortality and complications, reflecting the severe and complex nature of neural tissue damage (Jeong et al., 2024; Kumar, 2022).

Spinal cord lesion length was identified as a crucial negative prognostic factor in our analysis, reinforcing findings from prior studies (Kamal et al., 2023; Aarabi et al., 2017; Schading-Sassenhausen et al., 2024). Raj Kamal et al. reported that patients with length of spinal cord lesion less than 30 mm had a greater likelihood of achieving grade conversion, regardless of surgical timing (Kamal et al., 2023). Longer lesions are indicative of severe primary and secondary injury processes, including enhanced neuronal apoptosis, demyelination, and disruption of neural connectivity, all of which limit the potential for neurological recovery (Jaffer et al., 2023; Maugeri et al., 2023; Garifulin et al., 2024; Lebret et al., 2025).

Severe spinal canal stenosis post-injury independently predicted poorer neurological recovery, a finding consistent with earlier studies (Bimantara et al., 2022; Pascal-Moussellard et al., 2005). Bimantara et al. highlighted that severe lumbar spinal stenosis independently predicted poorer 8-week neurogenic claudication outcome scores post-decompression-stabilization-fusion (Bimantara et al., 2022). The hypothesis proposed is that severe stenosis exacerbates ischemia, edema, and inflammation, which further perpetuate the cycle of secondary spinal cord injury and hinder recovery (Cho et al., 2017; Amar, 2007).

Our analysis also revealed that prolonged ICU stays were significantly associated with worse neurological outcomes. This finding is in line with previous research indicating that extended ICU stays correlate with poorer functional outcomes, due to increased systemic complications such as infections, thromboembolic events, pulmonary issues, pressure ulcers, and delays in initiating rehabilitation (Gour-Provencal et al., 2021; Kopp et al., 2017; Weiterer et al., 2019; Schreiber et al., 2023; Ko and Ko, 2022). These complications often lead to longer periods of immobilization, which directly affect both neurological and functional recovery trajectories.

Interestingly, initial AIS grade and T2WI hyperintensity were not identified as independent predictors of neurological recovery in our cohort. While these markers remain clinically valuable for early neurological assessment and classification, their prognostic significance may be diminished when structural factors such as lesion length and spinal canal stenosis are taken into account. This finding contrasts with previous studies, including those by Stenimahitis et al. and Dobran et al. (Stenimahitis et al., 2024; Dobran et al., 2023). Such discrepancies are likely attributable to differences in follow-up duration, patient population characteristics, and analytical approaches. Our findings suggest that although clinical and imaging indicators both play important roles, structural MRI parameters may offer more consistent prognostic value in multivariate analyses.

### 4.3 Impact of neuropathic pain and bowel/bladder dysfunction on quality of life Post-SCI

Neuropathic pain significantly impairs motor function, the ability to perform activities of daily living and quality of life, as highlighted in the previous studies (Khan et al., 2022; Nees et al., 2017). The authors suggest that chronic pain often leads to dependency on caregivers, limiting the patient's participation in rehabilitation programs and hindering independence and quality of life. Our study also found that patients with no neurological recovery had significantly higher rates of neuropathic pain compared to those with neurological recovery, further emphasizing the negative impact of neuropathic pain on rehabilitation outcomes.

Bowel/bladder dysfunction were associated with lower quality of life post-SCI, consistent with existing literature (Stenimahitis et al., 2024; Ture et al., 2023; Liu et al., 2009). Stenimahitis et al. reported that reductions in bowel and bladder dysfunction correlated with improvements in AIS scores over a median follow-up of 3.7 years (Stenimahitis et al., 2024). Functional independence in bowel and bladder management greatly enhances quality of life, reduces complications such as urinary tract infections, and facilitates better social reintegration and psychological well-being post-injury.

### 4.4 Clinical implications and rehabilitation strategies

Clinically, our findings emphasize timely decompression surgery, precise injury assessment, strategic ICU management, and tailored interventions addressing bowel/bladder dysfunction and neuropathic pain management to optimize patient recovery. Integration of multidisciplinary rehabilitation approaches, including early physical therapy, psychological support, pain management, and patient education, should be emphasized to enhance comprehensive outcomes.

### 4.5 Strengths and limitations

Strengths of this study include rigorous statistical analyses and detailed clinical data collection, providing robust insights into predictive factors influencing neurological outcomes. However, it is important to note the limitations in our study such as the retrospective study design, single-center data collection, potential selection biases, and the lack of comprehensive psychosocial and comorbidity assessments.

## 5 Conclusion

In conclusion, the results of this study demonstrate that early surgical decompression, careful assessment of injury severity, lesion characteristics, spinal canal stenosis, and optimal ICU management are significantly associated with neurological recovery following traumatic cervical spinal cord injury (SCI). These findings highlight the importance of individualized management strategies in clinical practice to optimize neurological recovery in patients with traumatic cervical SCI.

## Informed consent statement

N/A.

## Authors' contributions

• Ngo Dinh Trung: Conception and design of the study, analysis and interpretation of data, drafting the manuscript, revising it critically for important intellectual content.

• Hoang Xuan Su: Acquisition of data, analysis and interpretation of data, revising the manuscript critically for important intellectual content.

• Dao Trong Chinh: Acquisition of data, analysis of data, revising the manuscript for intellectual content.

• Nguyen Chi Tam: Analysis and interpretation of data, drafting the manuscript, revising it critically.

• Le Nam Khanh: Statistical analysis and interpretation of results, revising the manuscript for intellectual content.

• Do Van Nam: Corresponding author, coordination of the study, manuscript revisions.

All authors have read and approved the final version of the manuscript and agree to be accountable for all aspects of the work.

## Funding

This research did not receive any specific grant from funding agencies in the public, commercial, or not-for-profit sectors.

## Declaration of competing interest

The authors declare that they have no known competing financial interests or personal relationships that could have appeared to influence the work reported in this paper.
